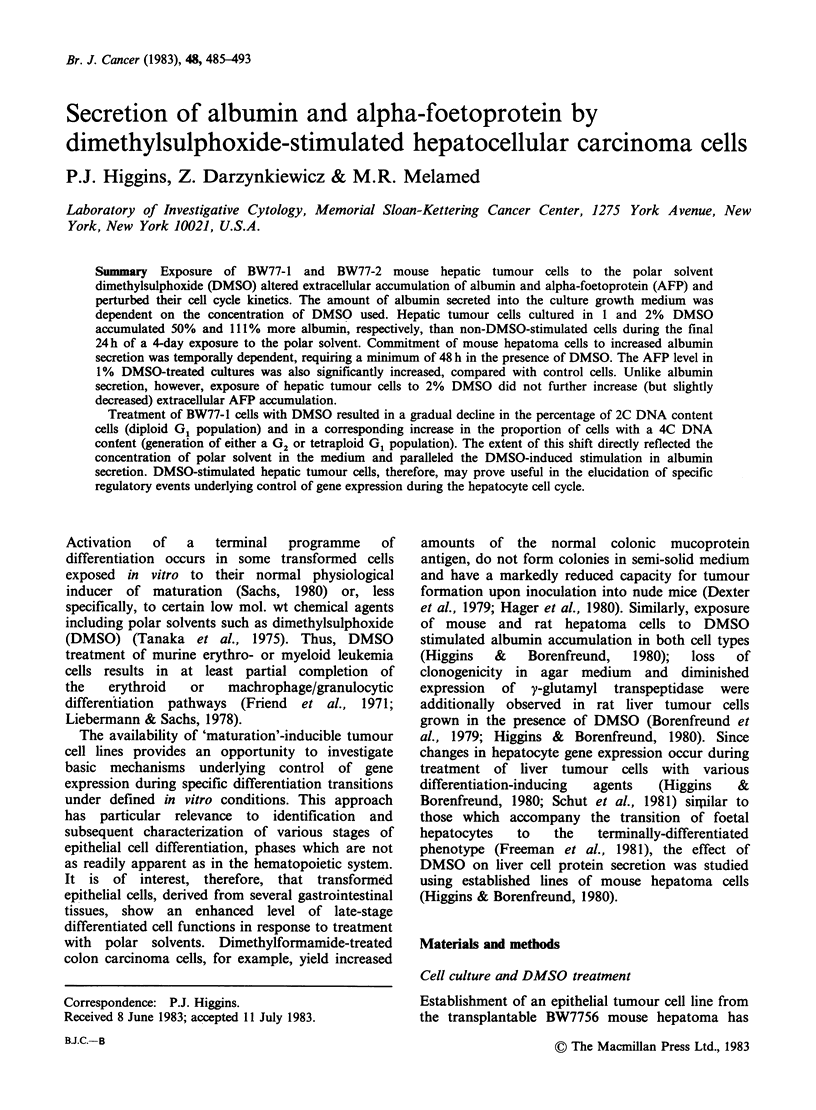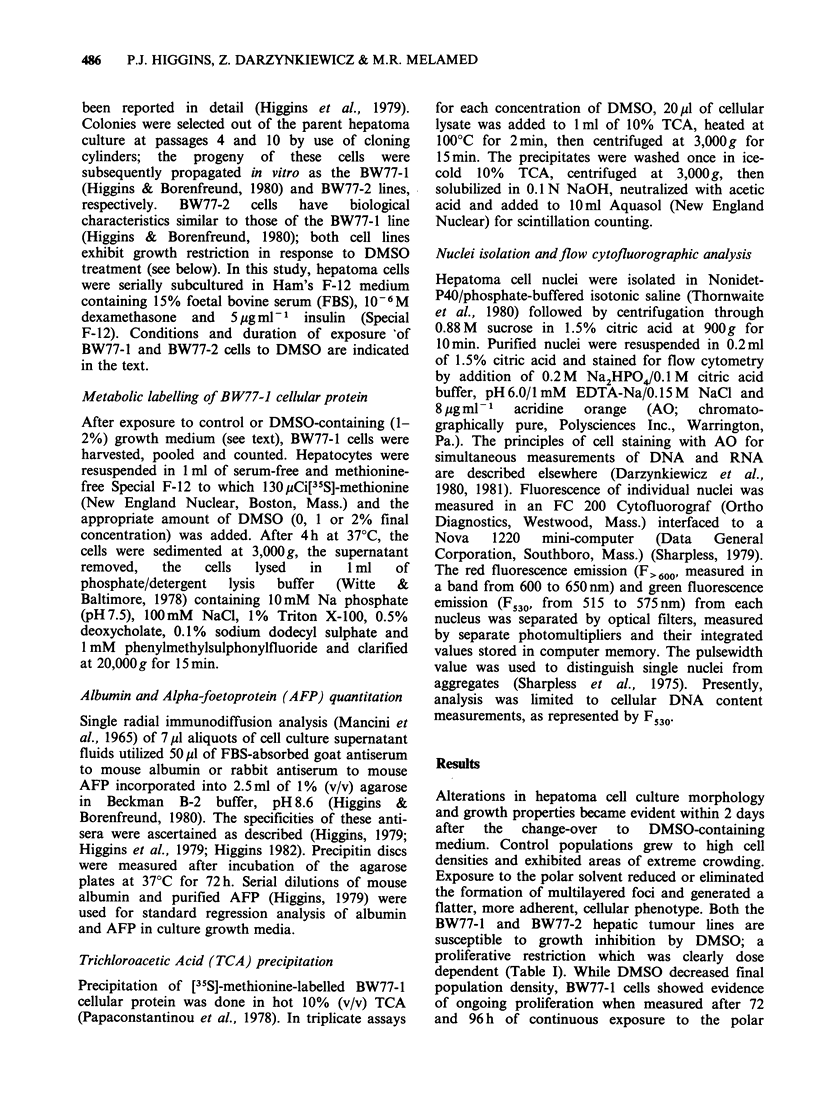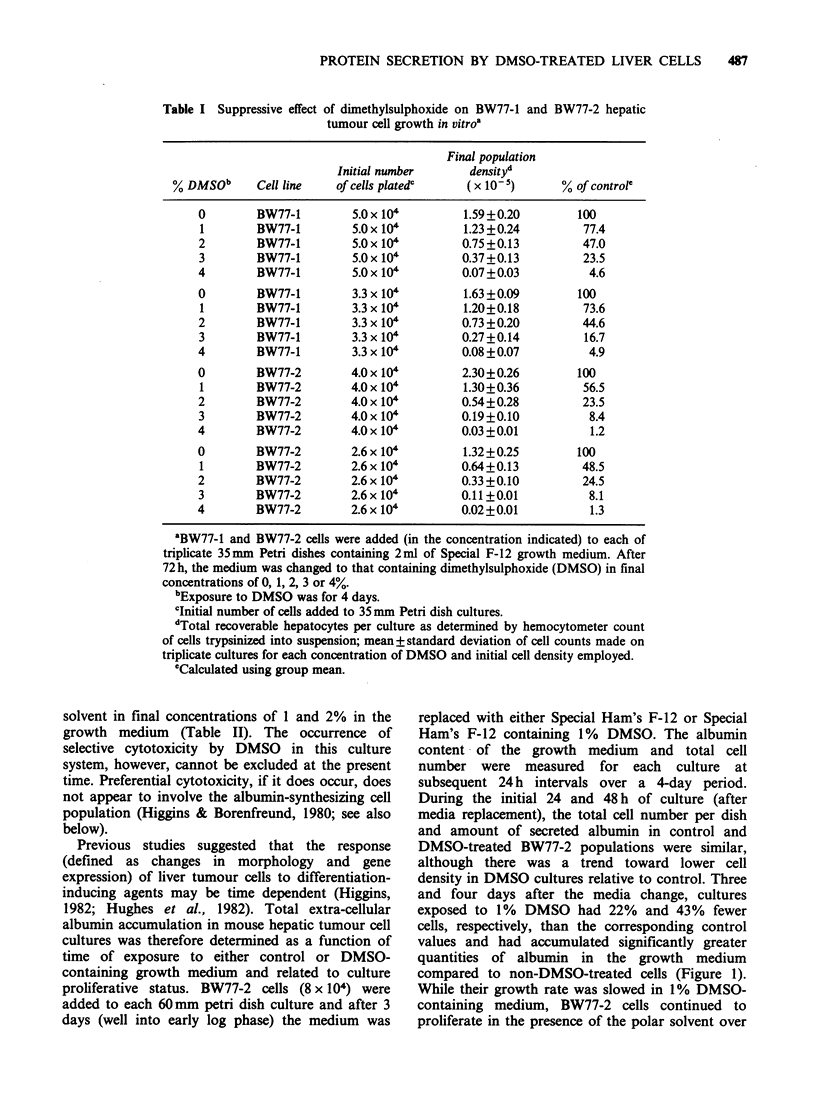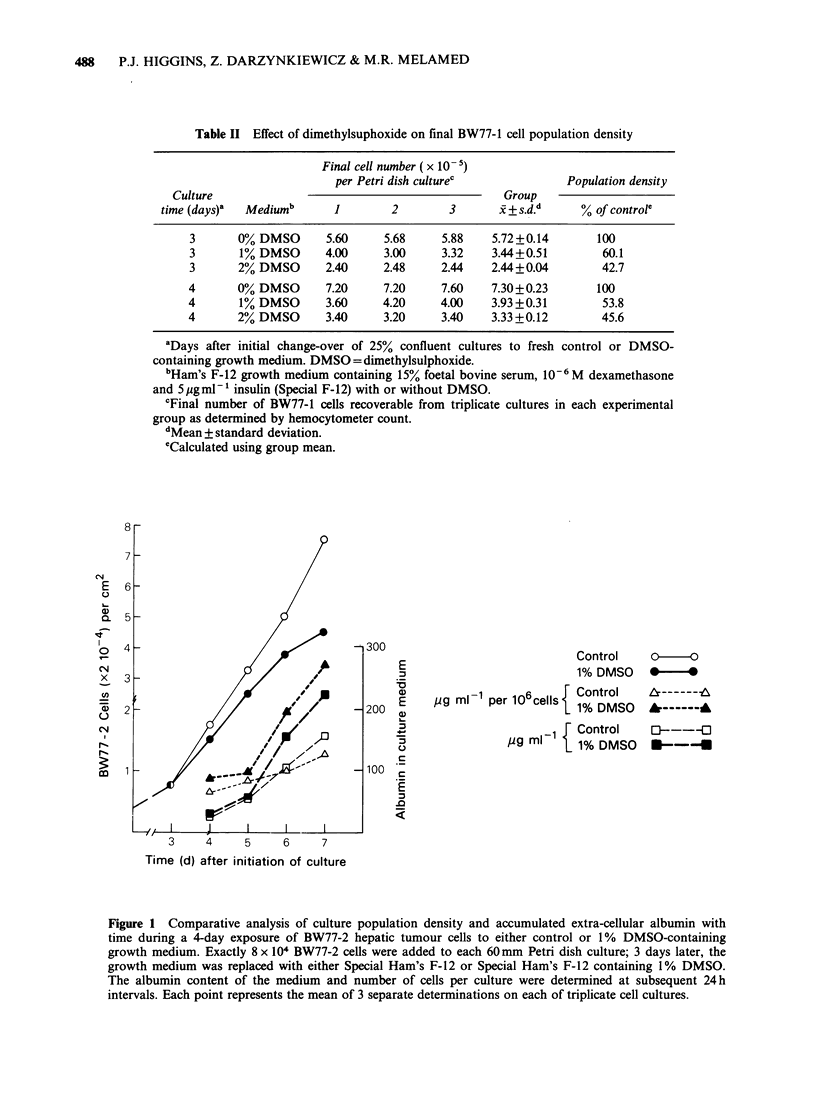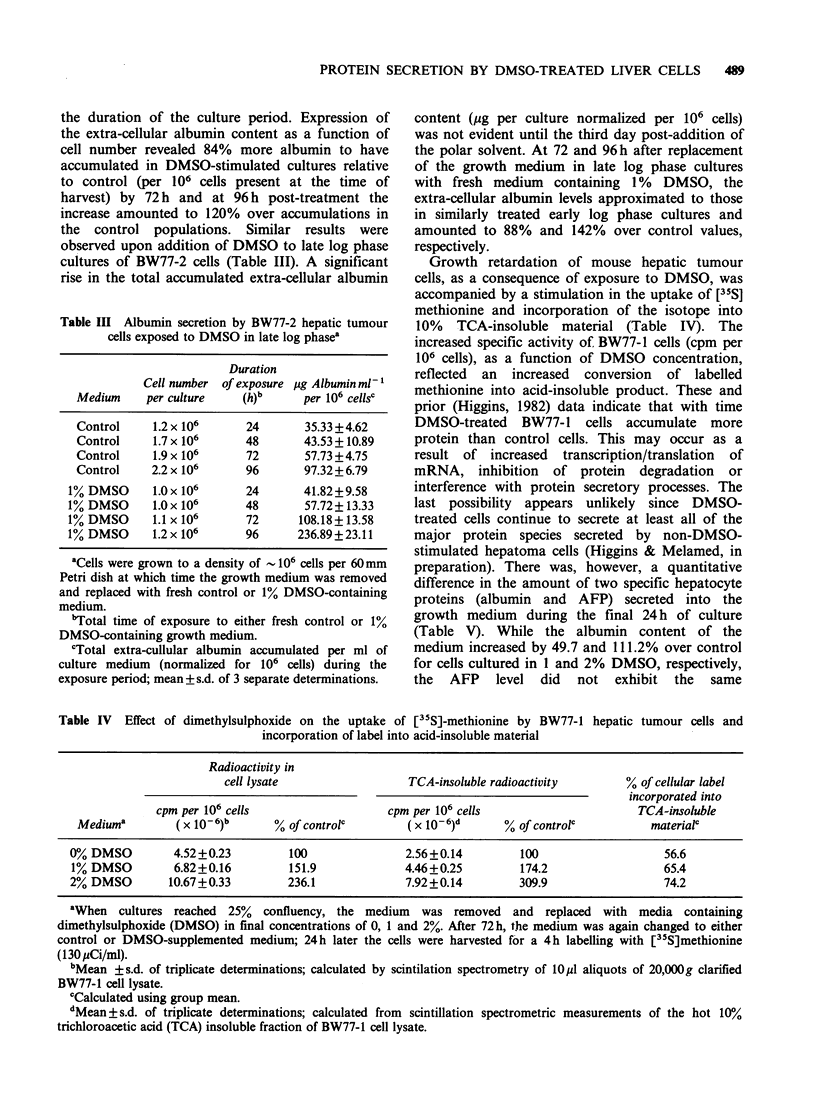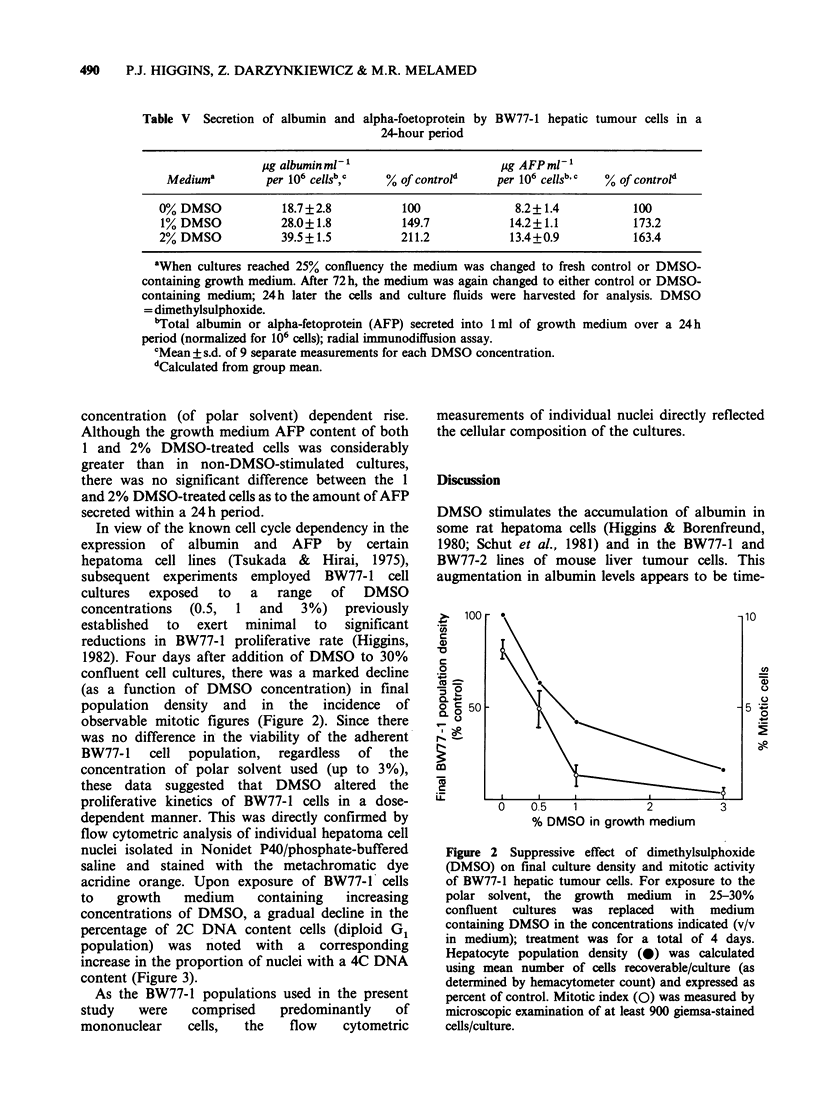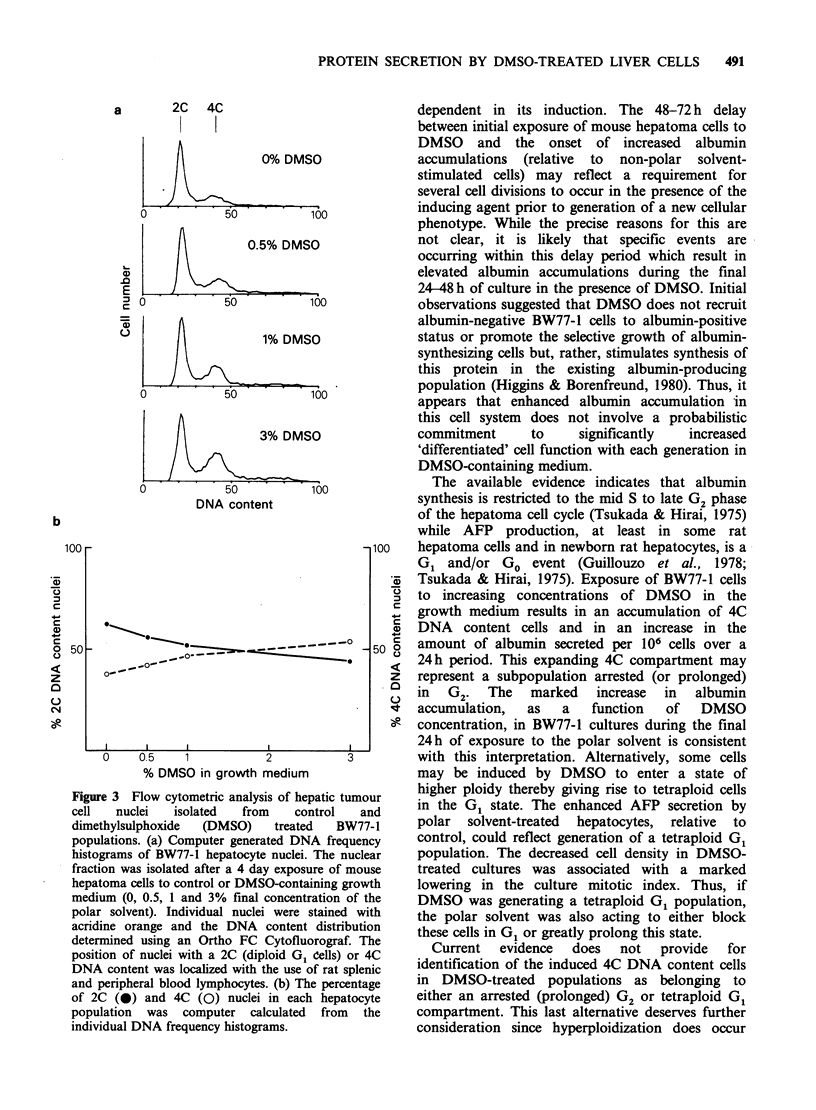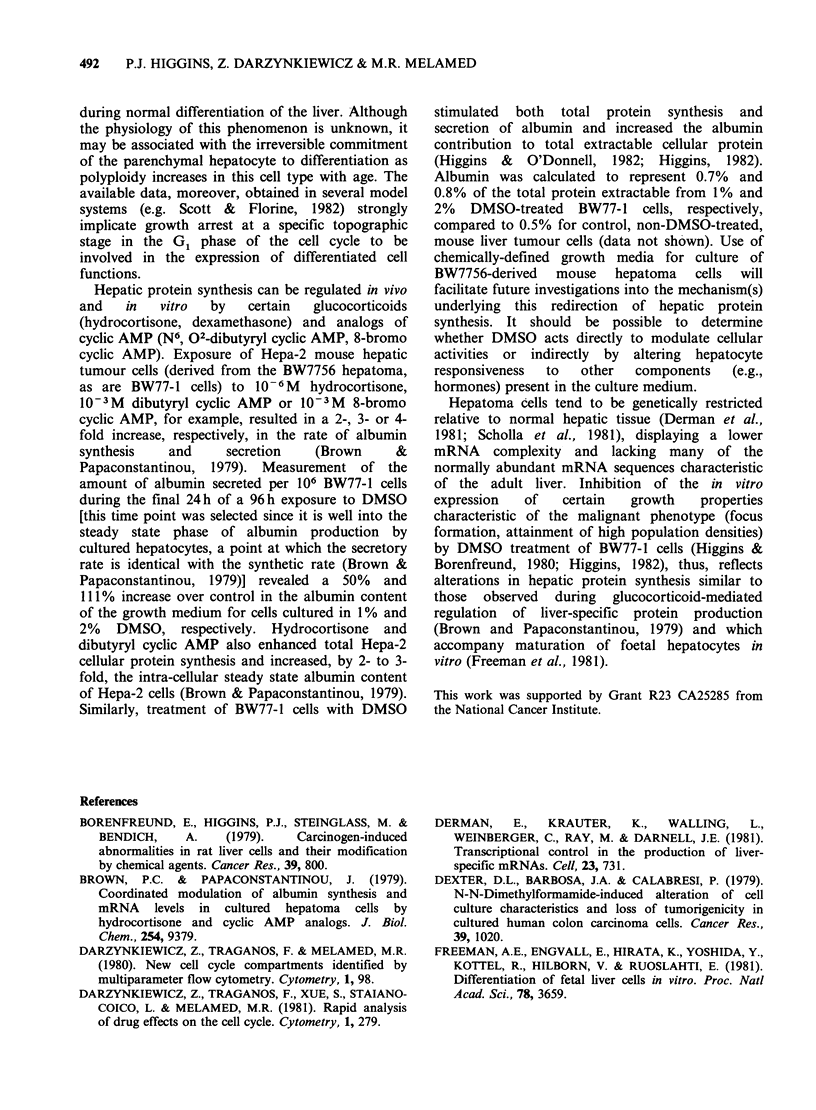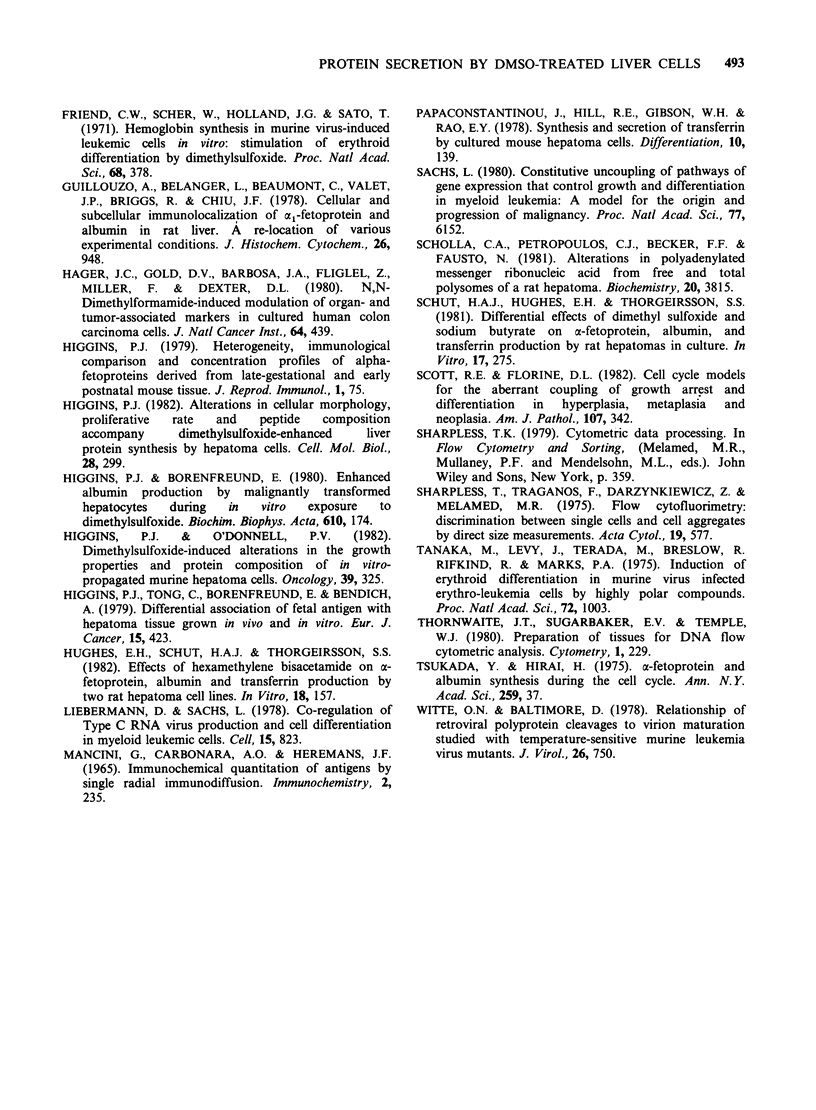# Secretion of albumin and alpha-foetoprotein by dimethylsulphoxide-stimulated hepatocellular carcinoma cells.

**DOI:** 10.1038/bjc.1983.221

**Published:** 1983-10

**Authors:** P. J. Higgins, Z. Darzynkiewicz, M. R. Melamed

## Abstract

Exposure of BW77-1 and BW77-2 mouse hepatic tumour cells to the polar solvent dimethylsulphoxide (DMSO) altered extracellular accumulation of albumin and alpha-foetoprotein (AFP) and perturbed their cell cycle kinetics. The amount of albumin secreted into the culture growth medium was dependent on the concentration of DMSO used. Hepatic tumour cells cultured in 1 and 2% DMSO accumulated 50% and 111% more albumin, respectively, than non-DMSO-stimulated cells during the final 24 h of a 4-day exposure to the polar solvent. Commitment of mouse hepatoma cells to increased albumin secretion was temporally dependent, requiring a minimum of 48 h in the presence of DMSO. The AFP level in 1% DMSO-treated cultures was also significantly increased, compared with control cells. Unlike albumin secretion, however, exposure of hepatic tumour cells to 2% DMSO did not further increase (but slightly decreased) extracellular AFP accumulation. Treatment of BW77-1 cells with DMSO resulted in a gradual decline in the percentage of 2C DNA content cells (diploid G1 population) and in a corresponding increase in the proportion of cells with a 4C DNA content (generation of either a G2 or tetraploid G1 population). The extent of this shift directly reflected the concentration of polar solvent in the medium and paralleled the DMSO-induced stimulation in albumin secretion. DMSO-stimulated hepatic tumour cells, therefore, may prove useful in the elucidation of specific regulatory events underlying control of gene expression during the hepatocyte cell cycle.


*Br. J. Cancer* (1983), **48**, 485–493

### Secretion of albumin and alpha-foetoprotein by dimethylsulphoxide-stimulated hepatocellular carcinoma cells

P.J. Higgins, Z. Darzynkiewicz & M.R. Melamed

*Laboratory of Investigative Cytology, Memorial Sloan-Kettering Cancer Center, 1275 York Avenue, New York, New York 10021, U.S.A.*

**Summary** Exposure of BW77-1 and BW77-2 mouse hepatic tumour cells to the polar solvent dimethylsulphoxide (DMSO) altered extracellular accumulation of albumin and alpha-foetoprotein (AFP) and perturbed their cell cycle kinetics. The amount of albumin secreted into the culture growth medium was dependent on the concentration of DMSO used. Hepatic tumour cells cultured in 1 and 2% DMSO accumulated 50% and 111% more albumin, respectively, than non-DMSO-stimulated cells during the final 24 h of a 4-day exposure to the polar solvent. Commitment of mouse hepatoma cells to increased albumin secretion was temporally dependent, requiring a minimum of 48 h in the presence of DMSO. The AFP level in 1% DMSO-treated cultures was also significantly increased, compared with control cells. Unlike albumin secretion, however, exposure of hepatic tumour cells to 2% DMSO did not further increase (but slightly decreased) extracellular AFP accumulation.

Treatment of BW77-1 cells with DMSO resulted in a gradual decline in the percentage of 2C DNA content cells (diploid $G_1$ population) and in a corresponding increase in the proportion of cells with a 4C DNA content (generation of either a $G_2$ or tetraploid $G_1$ population). The extent of this shift directly reflected the concentration of polar solvent in the medium and paralleled the DMSO-induced stimulation in albumin secretion. DMSO-stimulated hepatic tumour cells, therefore, may prove useful in the elucidation of specific regulatory events underlying control of gene expression during the hepatocyte cell cycle.

Activation of a terminal programme of differentiation occurs in some transformed cells exposed *in vitro* to their normal physiological inducer of maturation (Sachs, 1980) or, less specifically, to certain low mol. wt chemical agents including polar solvents such as dimethylsulphoxide (DMSO) (Tanaka *et al.*, 1975). Thus, DMSO treatment of murine erythro- or myeloid leukemia cells results in at least partial completion of the erythroid or macrophage/granulocytic differentiation pathways (Friend *et al.*, 1971; Liebermann & Sachs, 1978).

The availability of 'maturation'-inducible tumour cell lines provides an opportunity to investigate basic mechanisms underlying control of gene expression during specific differentiation transitions under defined *in vitro* conditions. This approach has particular relevance to identification and subsequent characterization of various stages of epithelial cell differentiation, phases which are not as readily apparent as in the hematopoietic system. It is of interest, therefore, that transformed epithelial cells, derived from several gastrointestinal tissues, show an enhanced level of late-stage differentiated cell functions in response to treatment with polar solvents. Dimethylformamide-treated colon carcinoma cells, for example, yield increased amounts of the normal colonic mucoprotein antigen, do not form colonies in semi-solid medium and have a markedly reduced capacity for tumour formation upon inoculation into nude mice (Dexter *et al.*, 1979; Hager *et al.*, 1980). Similarly, exposure of mouse and rat hepatoma cells to DMSO stimulated albumin accumulation in both cell types (Higgins & Borenfreund, 1980); loss of clonogenicity in agar medium and diminished expression of γ-glutamyl transpeptidase were additionally observed in rat liver tumour cells grown in the presence of DMSO (Borenfreund *et al.*, 1979; Higgins & Borenfreund, 1980). Since changes in hepatocyte gene expression occur during treatment of liver tumour cells with various differentiation-inducing agents (Higgins & Borenfreund, 1980; Schut *et al.*, 1981) similar to those which accompany the transition of foetal hepatocytes to the terminally-differentiated phenotype (Freeman *et al.*, 1981), the effect of DMSO on liver cell protein secretion was studied using established lines of mouse hepatoma cells (Higgins & Borenfreund, 1980).

### Materials and methods

### *Cell culture and DMSO treatment*

Establishment of an epithelial tumour cell line from the transplantable BW7756 mouse hepatoma has

Correspondence: P.J. Higgins.
Received 8 June 1983; accepted 11 July 1983.

© The Macmillan Press Ltd., 1983

been reported in detail (Higgins *et al.*, 1979). Colonies were selected out of the parent hepatoma culture at passages 4 and 10 by use of cloning cylinders; the progeny of these cells were subsequently propagated *in vitro* as the BW77-1 (Higgins & Borenfreund, 1980) and BW77-2 lines, respectively. BW77-2 cells have biological characteristics similar to those of the BW77-1 line (Higgins & Borenfreund, 1980); both cell lines exhibit growth restriction in response to DMSO treatment (see below). In this study, hepatoma cells were serially subcultured in Ham's F-12 medium containing 15% foetal bovine serum (FBS), $10^{-6}$M dexamethasone and 5 $\mu$g ml$^{-1}$ insulin (Special F-12). Conditions and duration of exposure of BW77-1 and BW77-2 cells to DMSO are indicated in the text.

*Metabolic labelling of BW77-1 cellular protein*

After exposure to control or DMSO-containing (1–2%) growth medium (see text), BW77-1 cells were harvested, pooled and counted. Hepatocytes were resuspended in 1 ml of serum-free and methionine-free Special F-12 to which 130 $\mu$Ci[$^{35}$S]-methionine (New England Nuclear, Boston, Mass.) and the appropriate amount of DMSO (0, 1 or 2% final concentration) was added. After 4 h at 37°C, the cells were sedimented at 3,000 *g*, the supernatant removed, the cells lysed in 1 ml of phosphate/detergent lysis buffer (Witte & Baltimore, 1978) containing 10 mM Na phosphate (pH 7.5), 100 mM NaCl, 1% Triton X-100, 0.5% deoxycholate, 0.1% sodium dodecyl sulphate and 1 mM phenylmethylsulphonylfluoride and clarified at 20,000 *g* for 15 min.

*Albumin and Alpha-foetoprotein (AFP) quantitation*

Single radial immunodiffusion analysis (Mancini *et al.*, 1965) of 7 $\mu$l aliquots of cell culture supernatant fluids utilized 50 $\mu$l of FBS-absorbed goat antiserum to mouse albumin or rabbit antiserum to mouse AFP incorporated into 2.5 ml of 1% (v/v) agarose in Beckman B-2 buffer, pH 8.6 (Higgins & Borenfreund, 1980). The specificities of these antisera were ascertained as described (Higgins, 1979; Higgins *et al.*, 1979; Higgins 1982). Precipitin discs were measured after incubation of the agarose plates at 37°C for 72 h. Serial dilutions of mouse albumin and purified AFP (Higgins, 1979) were used for standard regression analysis of albumin and AFP in culture growth media.

*Trichloroacetic Acid (TCA) precipitation*

Precipitation of [$^{35}$S]-methionine-labelled BW77-1 cellular protein was done in hot 10% (v/v) TCA (Papaconstantinou *et al.*, 1978). In triplicate assays for each concentration of DMSO, 20 $\mu$l of cellular lysate was added to 1 ml of 10% TCA, heated at 100°C for 2 min, then centrifuged at 3,000 *g* for 15 min. The precipitates were washed once in ice-cold 10% TCA, centrifuged at 3,000 *g*, then solubilized in 0.1 N NaOH, neutralized with acetic acid and added to 10 ml Aquasol (New England Nuclear) for scintillation counting.

*Nuclei isolation and flow cytofluorographic analysis*

Hepatoma cell nuclei were isolated in Nonidet-P40/phosphate-buffered isotonic saline (Thornwaite *et al.*, 1980) followed by centrifugation through 0.88 M sucrose in 1.5% citric acid at 900 *g* for 10 min. Purified nuclei were resuspended in 0.2 ml of 1.5% citric acid and stained for flow cytometry by addition of 0.2 M $Na_2HPO_4$/0.1 M citric acid buffer, pH 6.0/1 mM EDTA-Na/0.15 M NaCl and 8 $\mu$g ml$^{-1}$ acridine orange (AO; chromatographically pure, Polysciences Inc., Warrington, Pa.). The principles of cell staining with AO for simultaneous measurements of DNA and RNA are described elsewhere (Darzynkiewicz *et al.*, 1980, 1981). Fluorescence of individual nuclei was measured in an FC 200 Cytofluorograf (Ortho Diagnostics, Westwood, Mass.) interfaced to a Nova 1220 mini-computer (Data General Corporation, Southboro, Mass.) (Sharpless, 1979). The red fluorescence emission ($F_{>600}$, measured in a band from 600 to 650 nm) and green fluorescence emission ($F_{530}$, from 515 to 575 nm) from each nucleus was separated by optical filters, measured by separate photomultipliers and their integrated values stored in computer memory. The pulsewidth value was used to distinguish single nuclei from aggregates (Sharpless *et al.*, 1975). Presently, analysis was limited to cellular DNA content measurements, as represented by $F_{530}$.

### Results

Alterations in hepatoma cell culture morphology and growth properties became evident within 2 days after the change-over to DMSO-containing medium. Control populations grew to high cell densities and exhibited areas of extreme crowding. Exposure to the polar solvent reduced or eliminated the formation of multilayered foci and generated a flatter, more adherent, cellular phenotype. Both the BW77-1 and BW77-2 hepatic tumour lines are susceptible to growth inhibition by DMSO; a proliferative restriction which was clearly dose dependent (Table I). While DMSO decreased final population density, BW77-1 cells showed evidence of ongoing proliferation when measured after 72 and 96 h of continuous exposure to the polar

**Table I** Suppressive effect of dimethylsulphoxide on BW77-1 and BW77-2 hepatic tumour cell growth *in vitro*[a]

| *% DMSO*[b] | *Cell line* | *Initial number of cells plated*[c] | *Final population density*[d] ($\times 10^{-5}$) | *% of control*[e] |
|---|---|---|---|---|
| 0 | BW77-1 | $5.0\times10^4$ | $1.59\pm0.20$ | 100 |
| 1 | BW77-1 | $5.0\times10^4$ | $1.23\pm0.24$ | 77.4 |
| 2 | BW77-1 | $5.0\times10^4$ | $0.75\pm0.13$ | 47.0 |
| 3 | BW77-1 | $5.0\times10^4$ | $0.37\pm0.13$ | 23.5 |
| 4 | BW77-1 | $5.0\times10^4$ | $0.07\pm0.03$ | 4.6 |
| 0 | BW77-1 | $3.3\times10^4$ | $1.63\pm0.09$ | 100 |
| 1 | BW77-1 | $3.3\times10^4$ | $1.20\pm0.18$ | 73.6 |
| 2 | BW77-1 | $3.3\times10^4$ | $0.73\pm0.20$ | 44.6 |
| 3 | BW77-1 | $3.3\times10^4$ | $0.27\pm0.14$ | 16.7 |
| 4 | BW77-1 | $3.3\times10^4$ | $0.08\pm0.07$ | 4.9 |
| 0 | BW77-2 | $4.0\times10^4$ | $2.30\pm0.26$ | 100 |
| 1 | BW77-2 | $4.0\times10^4$ | $1.30\pm0.36$ | 56.5 |
| 2 | BW77-2 | $4.0\times10^4$ | $0.54\pm0.28$ | 23.5 |
| 3 | BW77-2 | $4.0\times10^4$ | $0.19\pm0.10$ | 8.4 |
| 4 | BW77-2 | $4.0\times10^4$ | $0.03\pm0.01$ | 1.2 |
| 0 | BW77-2 | $2.6\times10^4$ | $1.32\pm0.25$ | 100 |
| 1 | BW77-2 | $2.6\times10^4$ | $0.64\pm0.13$ | 48.5 |
| 2 | BW77-2 | $2.6\times10^4$ | $0.33\pm0.10$ | 24.5 |
| 3 | BW77-2 | $2.6\times10^4$ | $0.11\pm0.01$ | 8.1 |
| 4 | BW77-2 | $2.6\times10^4$ | $0.02\pm0.01$ | 1.3 |

[a]BW77-1 and BW77-2 cells were added (in the concentration indicated) to each of triplicate 35 mm Petri dishes containing 2 ml of Special F-12 growth medium. After 72 h, the medium was changed to that containing dimethylsulphoxide (DMSO) in final concentrations of 0, 1, 2, 3 or 4%.

[b]Exposure to DMSO was for 4 days.

[c]Initial number of cells added to 35 mm Petri dish cultures.

[d]Total recoverable hepatocytes per culture as determined by hemocytometer count of cells trypsinized into suspension; mean ± standard deviation of cell counts made on triplicate cultures for each concentration of DMSO and initial cell density employed.

[e]Calculated using group mean.

solvent in final concentrations of 1 and 2% in the growth medium (Table II). The occurrence of selective cytotoxicity by DMSO in this culture system, however, cannot be excluded at the present time. Preferential cytotoxicity, if it does occur, does not appear to involve the albumin-synthesizing cell population (Higgins & Borenfreund, 1980; see also below).

Previous studies suggested that the response (defined as changes in morphology and gene expression) of liver tumour cells to differentiation-inducing agents may be time dependent (Higgins, 1982; Hughes *et al.*, 1982). Total extra-cellular albumin accumulation in mouse hepatic tumour cell cultures was therefore determined as a function of time of exposure to either control or DMSO-containing growth medium and related to culture proliferative status. BW77-2 cells ($8\times10^4$) were added to each 60 mm petri dish culture and after 3 days (well into early log phase) the medium was replaced with either Special Ham's F-12 or Special Ham's F-12 containing 1% DMSO. The albumin content of the growth medium and total cell number were measured for each culture at subsequent 24 h intervals over a 4-day period. During the initial 24 and 48 h of culture (after media replacement), the total cell number per dish and amount of secreted albumin in control and DMSO-treated BW77-2 populations were similar, although there was a trend toward lower cell density in DMSO cultures relative to control. Three and four days after the media change, cultures exposed to 1% DMSO had 22% and 43% fewer cells, respectively, than the corresponding control values and had accumulated significantly greater quantities of albumin in the growth medium compared to non-DMSO-treated cells (Figure 1). While their growth rate was slowed in 1% DMSO-containing medium, BW77-2 cells continued to proliferate in the presence of the polar solvent over

**Table II** Effect of dimethylsuphoxide on final BW77-1 cell population density

| *Culture time (days)*[a] | *Medium*[b] | *Final cell number* ( $\times 10^{-5}$) *per Petri dish culture*[c] 1 | 2 | 3 | *Group* $\bar{x} \pm s.d.$[d] | *Population density* % *of control*[e] |
|---|---|---|---|---|---|---|
| 3 | 0% DMSO | 5.60 | 5.68 | 5.88 | 5.72±0.14 | 100 |
| 3 | 1% DMSO | 4.00 | 3.00 | 3.32 | 3.44±0.51 | 60.1 |
| 3 | 2% DMSO | 2.40 | 2.48 | 2.44 | 2.44±0.04 | 42.7 |
| 4 | 0% DMSO | 7.20 | 7.20 | 7.60 | 7.30±0.23 | 100 |
| 4 | 1% DMSO | 3.60 | 4.20 | 4.00 | 3.93±0.31 | 53.8 |
| 4 | 2% DMSO | 3.40 | 3.20 | 3.40 | 3.33±0.12 | 45.6 |

[a]Days after initial change-over of 25% confluent cultures to fresh control or DMSO-containing growth medium. DMSO = dimethylsulphoxide.

[b]Ham's F-12 growth medium containing 15% foetal bovine serum, $10^{-6}$ M dexamethasone and 5 $\mu g\,ml^{-1}$ insulin (Special F-12) with or without DMSO.

[c]Final number of BW77-1 cells recoverable from triplicate cultures in each experimental group as determined by hemocytometer count.

[d]Mean ± standard deviation.

[e]Calculated using group mean.

**Figure 1** Comparative analysis of culture population density and accumulated extra-cellular albumin with time during a 4-day exposure of BW77-2 hepatic tumour cells to either control or 1% DMSO-containing growth medium. Exactly $8 \times 10^4$ BW77-2 cells were added to each 60 mm Petri dish culture; 3 days later, the growth medium was replaced with either Special Ham's F-12 or Special Ham's F-12 containing 1% DMSO. The albumin content of the medium and number of cells per culture were determined at subsequent 24 h intervals. Each point represents the mean of 3 separate determinations on each of triplicate cell cultures.

the duration of the culture period. Expression of the extra-cellular albumin content as a function of cell number revealed 84% more albumin to have accumulated in DMSO-stimulated cultures relative to control (per $10^6$ cells present at the time of harvest) by 72 h and at 96 h post-treatment the increase amounted to 120% over accumulations in the control populations. Similar results were observed upon addition of DMSO to late log phase cultures of BW77-2 cells (Table III). A significant rise in the total accumulated extra-cellular albumin content (μg per culture normalized per $10^6$ cells) was not evident until the third day post-addition of the polar solvent. At 72 and 96 h after replacement of the growth medium in late log phase cultures with fresh medium containing 1% DMSO, the extra-cellular albumin levels approximated to those in similarly treated early log phase cultures and amounted to 88% and 142% over control values, respectively.

**Table III** Albumin secretion by BW77-2 hepatic tumour cells exposed to DMSO in late log phase[a]

| *Medium* | *Cell number per culture* | *Duration of exposure (h)*[b] | *μg Albumin ml$^{-1}$ per $10^6$ cells*[c] |
|---|---|---|---|
| Control | $1.2\times10^6$ | 24 | $35.33\pm4.62$ |
| Control | $1.7\times10^6$ | 48 | $43.53\pm10.89$ |
| Control | $1.9\times10^6$ | 72 | $57.73\pm4.75$ |
| Control | $2.2\times10^6$ | 96 | $97.32\pm6.79$ |
| 1% DMSO | $1.0\times10^6$ | 24 | $41.82\pm9.58$ |
| 1% DMSO | $1.0\times10^6$ | 48 | $57.72\pm13.33$ |
| 1% DMSO | $1.1\times10^6$ | 72 | $108.18\pm13.58$ |
| 1% DMSO | $1.2\times10^6$ | 96 | $236.89\pm23.11$ |

[a]Cells were grown to a density of $\sim10^6$ cells per 60 mm Petri dish at which time the growth medium was removed and replaced with fresh control or 1% DMSO-containing medium.

[b]Total time of exposure to either fresh control or 1% DMSO-containing growth medium.

[c]Total extra-cullular albumin accumulated per ml of culture medium (normalized for $10^6$ cells) during the exposure period; mean ± s.d. of 3 separate determinations.

Growth retardation of mouse hepatic tumour cells, as a consequence of exposure to DMSO, was accompanied by a stimulation in the uptake of [$^{35}$S] methionine and incorporation of the isotope into 10% TCA-insoluble material (Table IV). The increased specific activity of BW77-1 cells (cpm per $10^6$ cells), as a function of DMSO concentration, reflected an increased conversion of labelled methionine into acid-insoluble product. These and prior (Higgins, 1982) data indicate that with time DMSO-treated BW77-1 cells accumulate more protein than control cells. This may occur as a result of increased transcription/translation of mRNA, inhibition of protein degradation or interference with protein secretory processes. The last possibility appears unlikely since DMSO-treated cells continue to secrete at least all of the major protein species secreted by non-DMSO-stimulated hepatoma cells (Higgins & Melamed, in preparation). There was, however, a quantitative difference in the amount of two specific hepatocyte proteins (albumin and AFP) secreted into the growth medium during the final 24 h of culture (Table V). While the albumin content of the medium increased by 49.7 and 111.2% over control for cells cultured in 1 and 2% DMSO, respectively, the AFP level did not exhibit the same

**Table IV** Effect of dimethylsulphoxide on the uptake of [$^{35}$S]-methionine by BW77-1 hepatic tumour cells and incorporation of label into acid-insoluble material

| | *Radioactivity in cell lysate* | | *TCA-insoluble radioactivity* | | |
|---|---|---|---|---|---|
| *Medium*[a] | *cpm per $10^6$ cells ($\times10^{-6}$)*[b] | *% of control*[c] | *cpm per $10^6$ cells ($\times10^{-6}$)*[d] | *% of control*[c] | *% of cellular label incorporated into TCA-insoluble material*[c] |
| 0% DMSO | $4.52\pm0.23$ | 100 | $2.56\pm0.14$ | 100 | 56.6 |
| 1% DMSO | $6.82\pm0.16$ | 151.9 | $4.46\pm0.25$ | 174.2 | 65.4 |
| 2% DMSO | $10.67\pm0.33$ | 236.1 | $7.92\pm0.14$ | 309.9 | 74.2 |

[a]When cultures reached 25% confluency, the medium was removed and replaced with media containing dimethylsulphoxide (DMSO) in final concentrations of 0, 1 and 2%. After 72 h, the medium was again changed to either control or DMSO-supplemented medium; 24 h later the cells were harvested for a 4 h labelling with [$^{35}$S]methionine (130 μCi/ml).

[b]Mean ± s.d. of triplicate determinations; calculated by scintilation spectrometry of 10 μl aliquots of 20,000 *g* clarified BW77-1 cell lysate.

[c]Calculated using group mean.

[d]Mean ± s.d. of triplicate determinations; calculated from scintillation spectrometric measurements of the hot 10% trichloroacetic acid (TCA) insoluble fraction of BW77-1 cell lysate.

**Table V** Secretion of albumin and alpha-foetoprotein by BW77-1 hepatic tumour cells in a 24-hour period

| *Medium*[a] | *μg albumin ml*$^{-1}$ *per* $10^6$ *cells*[b,c] | *% of control*[d] | *μg AFP ml*$^{-1}$ *per* $10^6$ *cells*[b,c] | *% of control*[d] |
|---|---|---|---|---|
| 0% DMSO | 18.7±2.8 | 100 | 8.2±1.4 | 100 |
| 1% DMSO | 28.0±1.8 | 149.7 | 14.2±1.1 | 173.2 |
| 2% DMSO | 39.5±1.5 | 211.2 | 13.4±0.9 | 163.4 |

[a]When cultures reached 25% confluency the medium was changed to fresh control or DMSO-containing growth medium. After 72 h, the medium was again changed to either control or DMSO-containing medium; 24 h later the cells and culture fluids were harvested for analysis. DMSO = dimethylsulphoxide.

[b]Total albumin or alpha-fetoprotein (AFP) secreted into 1 ml of growth medium over a 24 h period (normalized for $10^6$ cells); radial immunodiffusion assay.

[c]Mean ± s.d. of 9 separate measurements for each DMSO concentration.

[d]Calculated from group mean.

concentration (of polar solvent) dependent rise. Although the growth medium AFP content of both 1 and 2% DMSO-treated cells was considerably greater than in non-DMSO-stimulated cultures, there was no significant difference between the 1 and 2% DMSO-treated cells as to the amount of AFP secreted within a 24 h period.

In view of the known cell cycle dependency in the expression of albumin and AFP by certain hepatoma cell lines (Tsukada & Hirai, 1975), subsequent experiments employed BW77-1 cell cultures exposed to a range of DMSO concentrations (0.5, 1 and 3%) previously established to exert minimal to significant reductions in BW77-1 proliferative rate (Higgins, 1982). Four days after addition of DMSO to 30% confluent cell cultures, there was a marked decline (as a function of DMSO concentration) in final population density and in the incidence of observable mitotic figures (Figure 2). Since there was no difference in the viability of the adherent BW77-1 cell population, regardless of the concentration of polar solvent used (up to 3%), these data suggested that DMSO altered the proliferative kinetics of BW77-1 cells in a dose-dependent manner. This was directly confirmed by flow cytometric analysis of individual hepatoma cell nuclei isolated in Nonidet P40/phosphate-buffered saline and stained with the metachromatic dye acridine orange. Upon exposure of BW77-1 cells to growth medium containing increasing concentrations of DMSO, a gradual decline in the percentage of 2C DNA content cells (diploid $G_1$ population) was noted with a corresponding increase in the proportion of nuclei with a 4C DNA content (Figure 3).

As the BW77-1 populations used in the present study were comprised predominantly of mononuclear cells, the flow cytometric measurements of individual nuclei directly reflected the cellular composition of the cultures.

### Discussion

DMSO stimulates the accumulation of albumin in some rat hepatoma cells (Higgins & Borenfreund, 1980; Schut *et al.*, 1981) and in the BW77-1 and BW77-2 lines of mouse liver tumour cells. This augmentation in albumin levels appears to be time-

**Figure 2** Suppressive effect of dimethylsulphoxide (DMSO) on final culture density and mitotic activity of BW77-1 hepatic tumour cells. For exposure to the polar solvent, the growth medium in 25–30% confluent cultures was replaced with medium containing DMSO in the concentrations indicated (v/v in medium); treatment was for a total of 4 days. Hepatocyte population density (●) was calculated using mean number of cells recoverable/culture (as determined by hemacytometer count) and expressed as percent of control. Mitotic index (○) was measured by microscopic examination of at least 900 giemsa-stained cells/culture.

**Figure 3** Flow cytometric analysis of hepatic tumour cell nuclei isolated from control and dimethylsulphoxide (DMSO) treated BW77-1 populations. (a) Computer generated DNA frequency histograms of BW77-1 hepatocyte nuclei. The nuclear fraction was isolated after a 4 day exposure of mouse hepatoma cells to control or DMSO-containing growth medium (0, 0.5, 1 and 3% final concentration of the polar solvent). Individual nuclei were stained with acridine orange and the DNA content distribution determined using an Ortho FC Cytofluorograf. The position of nuclei with a 2C (diploid $G_1$ cells) or 4C DNA content was localized with the use of rat splenic and peripheral blood lymphocytes. (b) The percentage of 2C (●) and 4C (○) nuclei in each hepatocyte population was computer calculated from the individual DNA frequency histograms.

dependent in its induction. The 48–72 h delay between initial exposure of mouse hepatoma cells to DMSO and the onset of increased albumin accumulations (relative to non-polar solvent-stimulated cells) may reflect a requirement for several cell divisions to occur in the presence of the inducing agent prior to generation of a new cellular phenotype. While the precise reasons for this are not clear, it is likely that specific events are occurring within this delay period which result in elevated albumin accumulations during the final 24–48 h of culture in the presence of DMSO. Initial observations suggested that DMSO does not recruit albumin-negative BW77-1 cells to albumin-positive status or promote the selective growth of albumin-synthesizing cells but, rather, stimulates synthesis of this protein in the existing albumin-producing population (Higgins & Borenfreund, 1980). Thus, it appears that enhanced albumin accumulation in this cell system does not involve a probabilistic commitment to significantly increased 'differentiated' cell function with each generation in DMSO-containing medium.

The available evidence indicates that albumin synthesis is restricted to the mid S to late $G_2$ phase of the hepatoma cell cycle (Tsukada & Hirai, 1975) while AFP production, at least in some rat hepatoma cells and in newborn rat hepatocytes, is a $G_1$ and/or $G_0$ event (Guillouzo *et al.,* 1978; Tsukada & Hirai, 1975). Exposure of BW77-1 cells to increasing concentrations of DMSO in the growth medium results in an accumulation of 4C DNA content cells and in an increase in the amount of albumin secreted per $10^6$ cells over a 24 h period. This expanding 4C compartment may represent a subpopulation arrested (or prolonged) in $G_2$. The marked increase in albumin accumulation, as a function of DMSO concentration, in BW77-1 cultures during the final 24 h of exposure to the polar solvent is consistent with this interpretation. Alternatively, some cells may be induced by DMSO to enter a state of higher ploidy thereby giving rise to tetraploid cells in the $G_1$ state. The enhanced AFP secretion by polar solvent-treated hepatocytes, relative to control, could reflect generation of a tetraploid $G_1$ population. The decreased cell density in DMSO-treated cultures was associated with a marked lowering in the culture mitotic index. Thus, if DMSO was generating a tetraploid $G_1$ population, the polar solvent was also acting to either block these cells in $G_1$ or greatly prolong this state.

Current evidence does not provide for identification of the induced 4C DNA content cells in DMSO-treated populations as belonging to either an arrested (prolonged) $G_2$ or tetraploid $G_1$ compartment. This last alternative deserves further consideration since hyperploidization does occur during normal differentiation of the liver. Although the physiology of this phenomenon is unknown, it may be associated with the irreversible commitment of the parenchymal hepatocyte to differentiation as polyploidy increases in this cell type with age. The available data, moreover, obtained in several model systems (e.g. Scott & Florine, 1982) strongly implicate growth arrest at a specific topographic stage in the $G_1$ phase of the cell cycle to be involved in the expression of differentiated cell functions.

Hepatic protein synthesis can be regulated *in vivo* and *in vitro* by certain glucocorticoids (hydrocortisone, dexamethasone) and analogs of cyclic AMP ($N^6$, $O^2$-dibutyryl cyclic AMP, 8-bromo cyclic AMP). Exposure of Hepa-2 mouse hepatic tumour cells (derived from the BW7756 hepatoma, as are BW77-1 cells) to $10^{-6}$M hydrocortisone, $10^{-3}$M dibutyryl cyclic AMP or $10^{-3}$M 8-bromo cyclic AMP, for example, resulted in a 2-, 3- or 4-fold increase, respectively, in the rate of albumin synthesis and secretion (Brown & Papaconstantinou, 1979). Measurement of the amount of albumin secreted per $10^6$ BW77-1 cells during the final 24 h of a 96 h exposure to DMSO [this time point was selected since it is well into the steady state phase of albumin production by cultured hepatocytes, a point at which the secretory rate is identical with the synthetic rate (Brown & Papaconstantinou, 1979)] revealed a 50% and 111% increase over control in the albumin content of the growth medium for cells cultured in 1% and 2% DMSO, respectively. Hydrocortisone and dibutyryl cyclic AMP also enhanced total Hepa-2 cellular protein synthesis and increased, by 2- to 3-fold, the intra-cellular steady state albumin content of Hepa-2 cells (Brown & Papaconstantinou, 1979). Similarly, treatment of BW77-1 cells with DMSO stimulated both total protein synthesis and secretion of albumin and increased the albumin contribution to total extractable cellular protein (Higgins & O'Donnell, 1982; Higgins, 1982). Albumin was calculated to represent 0.7% and 0.8% of the total protein extractable from 1% and 2% DMSO-treated BW77-1 cells, respectively, compared to 0.5% for control, non-DMSO-treated, mouse liver tumour cells (data not shown). Use of chemically-defined growth media for culture of BW7756-derived mouse hepatoma cells will facilitate future investigations into the mechanism(s) underlying this redirection of hepatic protein synthesis. It should be possible to determine whether DMSO acts directly to modulate cellular activities or indirectly by altering hepatocyte responsiveness to other components (e.g., hormones) present in the culture medium.

Hepatoma cells tend to be genetically restricted relative to normal hepatic tissue (Derman *et al.*, 1981; Scholla *et al.*, 1981), displaying a lower mRNA complexity and lacking many of the normally abundant mRNA sequences characteristic of the adult liver. Inhibition of the *in vitro* expression of certain growth properties characteristic of the malignant phenotype (focus formation, attainment of high population densities) by DMSO treatment of BW77-1 cells (Higgins & Borenfreund, 1980; Higgins, 1982), thus, reflects alterations in hepatic protein synthesis similar to those observed during glucocorticoid-mediated regulation of liver-specific protein production (Brown and Papaconstantinou, 1979) and which accompany maturation of foetal hepatocytes *in vitro* (Freeman *et al.*, 1981).

This work was supported by Grant R23 CA25285 from the National Cancer Institute.

### References

BORENFREUND, E., HIGGINS, P.J., STEINGLASS, M. & BENDICH, A. (1979). Carcinogen-induced abnormalities in rat liver cells and their modification by chemical agents. *Cancer Res.*, **39**, 800.

BROWN, P.C. & PAPACONSTANTINOU, J. (1979). Coordinated modulation of albumin synthesis and mRNA levels in cultured hepatoma cells by hydrocortisone and cyclic AMP analogs. *J. Biol. Chem.*, **254**, 9379.

DARZYNKIEWICZ, Z., TRAGANOS, F. & MELAMED, M.R. (1980). New cell cycle compartments identified by multiparameter flow cytometry. *Cytometry*, **1**, 98.

DARZYNKIEWICZ, Z., TRAGANOS, F., XUE, S., STAIANO-COICO, L. & MELAMED, M.R. (1981). Rapid analysis of drug effects on the cell cycle. *Cytometry*, **1**, 279.

DERMAN, E., KRAUTER, K., WALLING, L., WEINBERGER, C., RAY, M. & DARNELL, J.E. (1981). Transcriptional control in the production of liver-specific mRNAs. *Cell*, **23**, 731.

DEXTER, D.L., BARBOSA, J.A. & CALABRESI, P. (1979). N-N-Dimethylformamide-induced alteration of cell culture characteristics and loss of tumorigenicity in cultured human colon carcinoma cells. *Cancer Res.*, **39**, 1020.

FREEMAN, A.E., ENGVALL, E., HIRATA, K., YOSHIDA, Y., KOTTEL, R., HILBORN, V. & RUOSLAHTI, E. (1981). Differentiation of fetal liver cells *in vitro*. *Proc. Natl Acad. Sci.*, **78**, 3659.

FRIEND, C.W., SCHER, W., HOLLAND, J.G. & SATO, T. (1971). Hemoglobin synthesis in murine virus-induced leukemic cells *in vitro*: stimulation of erythroid differentiation by dimethylsulfoxide. *Proc. Natl Acad. Sci.*, **68**, 378.

GUILLOUZO, A., BELANGER, L., BEAUMONT, C., VALET, J.P., BRIGGS, R. & CHIU, J.F. (1978). Cellular and subcellular immunolocalization of $\alpha_1$-fetoprotein and albumin in rat liver. A re-location of various experimental conditions. *J. Histochem. Cytochem.*, **26**, 948.

HAGER, J.C., GOLD, D.V., BARBOSA, J.A., FLIGLEL, Z., MILLER, F. & DEXTER, D.L. (1980). N,N-Dimethylformamide-induced modulation of organ- and tumor-associated markers in cultured human colon carcinoma cells. *J. Natl Cancer Inst.*, **64**, 439.

HIGGINS, P.J. (1979). Heterogeneity, immunological comparison and concentration profiles of alpha-fetoproteins derived from late-gestational and early postnatal mouse tissue. *J. Reprod. Immunol.*, **1**, 75.

HIGGINS, P.J. (1982). Alterations in cellular morphology, proliferative rate and peptide composition accompany dimethylsulfoxide-enhanced liver protein synthesis by hepatoma cells. *Cell. Mol. Biol.*, **28**, 299.

HIGGINS, P.J. & BORENFREUND, E. (1980). Enhanced albumin production by malignantly transformed hepatocytes during *in vitro* exposure to dimethylsulfoxide. *Biochim. Biophys. Acta*, **610**, 174.

HIGGINS, P.J. & O'DONNELL, P.V. (1982). Dimethylsulfoxide-induced alterations in the growth properties and protein composition of *in vitro*-propagated murine hepatoma cells. *Oncology*, **39**, 325.

HIGGINS, P.J., TONG, C., BORENFREUND, E. & BENDICH, A. (1979). Differential association of fetal antigen with hepatoma tissue grown *in vivo* and *in vitro*. *Eur. J. Cancer*, **15**, 423.

HUGHES, E.H., SCHUT, H.A.J. & THORGEIRSSON, S.S. (1982). Effects of hexamethylene bisacetamide on $\alpha$-fetoprotein, albumin and transferrin production by two rat hepatoma cell lines. *In Vitro*, **18**, 157.

LIEBERMANN, D. & SACHS, L. (1978). Co-regulation of Type C RNA virus production and cell differentiation in myeloid leukemic cells. *Cell*, **15**, 823.

MANCINI, G., CARBONARA, A.O. & HEREMANS, J.F. (1965). Immunochemical quantitation of antigens by single radial immunodiffusion. *Immunochemistry*, **2**, 235.

PAPACONSTANTINOU, J., HILL, R.E., GIBSON, W.H. & RAO, E.Y. (1978). Synthesis and secretion of transferrin by cultured mouse hepatoma cells. *Differentiation*, **10**, 139.

SACHS, L. (1980). Constitutive uncoupling of pathways of gene expression that control growth and differentiation in myeloid leukemia: A model for the origin and progression of malignancy. *Proc. Natl Acad. Sci.*, **77**, 6152.

SCHOLLA, C.A., PETROPOULOS, C.J., BECKER, F.F. & FAUSTO, N. (1981). Alterations in polyadenylated messenger ribonucleic acid from free and total polysomes of a rat hepatoma. *Biochemistry*, **20**, 3815.

SCHUT, H.A.J., HUGHES, E.H. & THORGEIRSSON, S.S. (1981). Differential effects of dimethyl sulfoxide and sodium butyrate on $\alpha$-fetoprotein, albumin, and transferrin production by rat hepatomas in culture. *In Vitro*, **17**, 275.

SCOTT, R.E. & FLORINE, D.L. (1982). Cell cycle models for the aberrant coupling of growth arrest and differentiation in hyperplasia, metaplasia and neoplasia. *Am. J. Pathol.*, **107**, 342.

SHARPLESS, T.K. (1979). Cytometric data processing. In *Flow Cytometry and Sorting*, (Melamed, M.R., Mullaney, P.F. and Mendelsohn, M.L., eds.). John Wiley and Sons, New York, p. 359.

SHARPLESS, T., TRAGANOS, F., DARZYNKIEWICZ, Z. & MELAMED, M.R. (1975). Flow cytofluorimetry: discrimination between single cells and cell aggregates by direct size measurements. *Acta Cytol.*, **19**, 577.

TANAKA, M., LEVY, J., TERADA, M., BRESLOW, R. RIFKIND, R. & MARKS, P.A. (1975). Induction of erythroid differentiation in murine virus infected erythro-leukemia cells by highly polar compounds. *Proc. Natl Acad. Sci.*, **72**, 1003.

THORNWAITE, J.T., SUGARBAKER, E.V. & TEMPLE, W.J. (1980). Preparation of tissues for DNA flow cytometric analysis. *Cytometry*, **1**, 229.

TSUKADA, Y. & HIRAI, H. (1975). $\alpha$-fetoprotein and albumin synthesis during the cell cycle. *Ann. N.Y. Acad. Sci.*, **259**, 37.

WITTE, O.N. & BALTIMORE, D. (1978). Relationship of retroviral polyprotein cleavages to virion maturation studied with temperature-sensitive murine leukemia virus mutants. *J. Virol.*, **26**, 750.